# Acceptability of a Web-Based and Tailored Intervention for the Self-Management of Pain After Cardiac Surgery: The Perception of Women and Men

**DOI:** 10.2196/resprot.3175

**Published:** 2014-11-20

**Authors:** Geraldine Martorella, Céline Gélinas, Margaret Purden

**Affiliations:** ^1^Faculty of NursingUniversity of MontrealMontreal, QCCanada; ^2^Ingram School of NursingMcGill UniversityMontreal, QCCanada; ^3^Centre for Nursing Research and Lady Davis InstituteJewish General HospitalMontreal, QCCanada; ^4^Alan Edwards Centre for Research on PainMcGill UniversityMontreal, QCCanada

**Keywords:** pain, postoperative, surgery, cardiac, patient education, Internet, mixed method

## Abstract

**Background:**

Approximately two thirds of adults undergoing cardiac surgery suffer from moderate to severe postoperative pain. Assisting patients with pain management is therefore critical to prevent its negative consequences. Information technologies have become part of our lifestyle and can facilitate the implementation of interventions to manage pain in a busy care setting. A computer-tailored and Web-based intervention—referred to as SOUtien à L’AutoGEstion-Traitement-Assistance Virtuelle Infirmière-Enseignement (SOULAGE-TAVIE)—for the self-management of pain was developed. Findings from a previous pilot randomized controlled trial (RCT) provided some evidence of the feasibility and preliminary effectiveness of this intervention in decreasing pain interference with a few postoperative activities and by modulating pain beliefs and analgesic intake. However, its acceptability from the patient’s perspective remains unclear. Moreover, the proportion of women is much lower in the cardiac surgical population, making it difficult to detect differences in experiences between men and women.

**Objective:**

The objectives were (1) to describe SOULAGE-TAVIE’s acceptability from the perspective of adults experiencing pain after cardiac surgery and (2) to compare the perceptions of men and women.

**Methods:**

A mixed-method approach was used to capture the various attributes of patients’ perceptions of the intervention’s acceptability and to compare the perceptions of men and women. Quota samples of men (n=10; mean age 62.5 years, SD 7.3) and women (n=10; mean age 64.3 years, SD 10.7) who had cardiac surgery in the past month were invited to view the intervention, complete a brief questionnaire rating its acceptability, and then to discuss each component in a 60-minute, semistructured interview. Mann-Whitney U tests were used to compare groups. The transcripts were content analyzed to generate themes based on patients’ experiences with the intervention and reports of acceptability. The content of each category and subcategory were compared between men and women. Frequency counts were also done to validate the emergence of a difference between the 2 subgroups.

**Results:**

Participants perceived the intervention to be very acceptable in terms of content and format, and tended to describe awareness-raising and convenient support experiences. Women scored higher than men in terms of the intervention’s appropriateness (U=13.5, *P*=.008). They were willing to adhere to the intervention based on the importance and relevance of the advice provided, whereas men were more focused on the delivery mode and its flexibility.

**Conclusions:**

This study underlined the acceptability of computer tailoring and persuasive communication to modulate pain beliefs and attitudes in an acute care context. Both men and women appreciated the Web-based interface and general self-guided approach of the intervention. The delivery of SOULAGE-TAVIE across the continuum of care seems to be an interesting avenue to influence the transition from acute to chronic postoperative pain.

## Introduction

Approximately two thirds of adults undergoing cardiac surgery suffer from moderate to severe postoperative pain and 75% of adults suffer from pain when breathing and coughing for as long as 7 days after surgery [1-3]. Several studies also showed that intensity of acute postoperative pain predicted the presence and severity of pain after discharge and is a risk factor for the development of chronic postoperative pain (CPOP) [2-5]. Indeed, pain may become chronic in 17% to 56% of adults in the 2 years following cardiac surgery, potentially compromising their recovery and daily functioning [2,3,6,7]. These prevalence rates are substantial considering that cardiac surgeries rank among the most frequent surgical procedures [8]. Therefore, assisting patients with pain management is critical to prevent its negative consequences. Individual beliefs and attitudes regarding pain and its relief interfere with the communication of pain and its management, which could partially explain inadequate levels of analgesic consumption observed in many patients following cardiac surgery [9,10]. Computer-tailored and Web-based interventions have been recognized for their efficacy on information integration and behavioral change [11-14]. Information technologies have become part of our lifestyle and can facilitate the implementation of interventions influencing pain management in a busy care setting.

**Figure 1:**
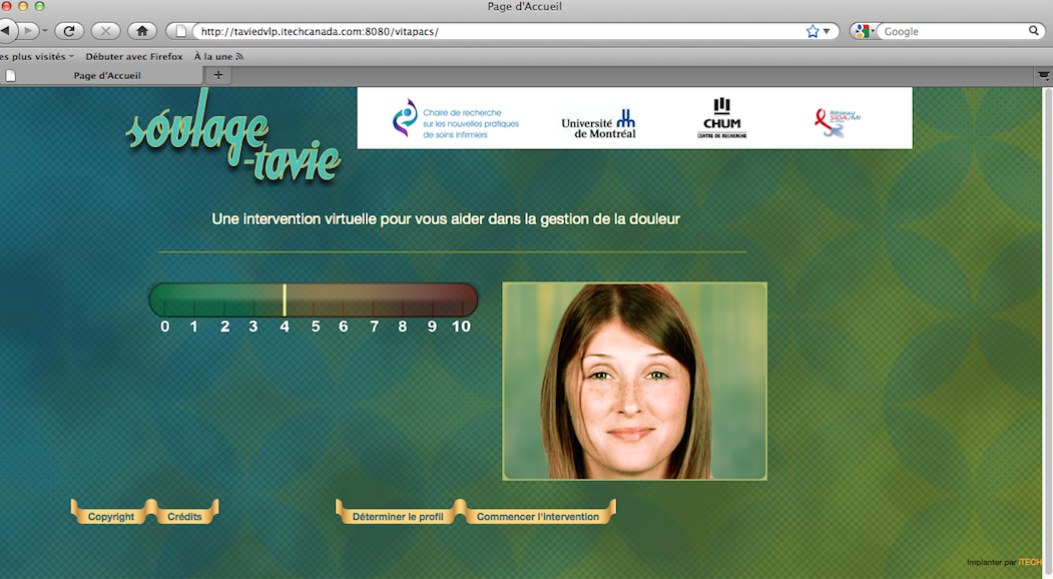
Homepage of the SOULAGE-TAVIE website showing functions to determine patient profile and to start the intervention.

**Figure 2:**
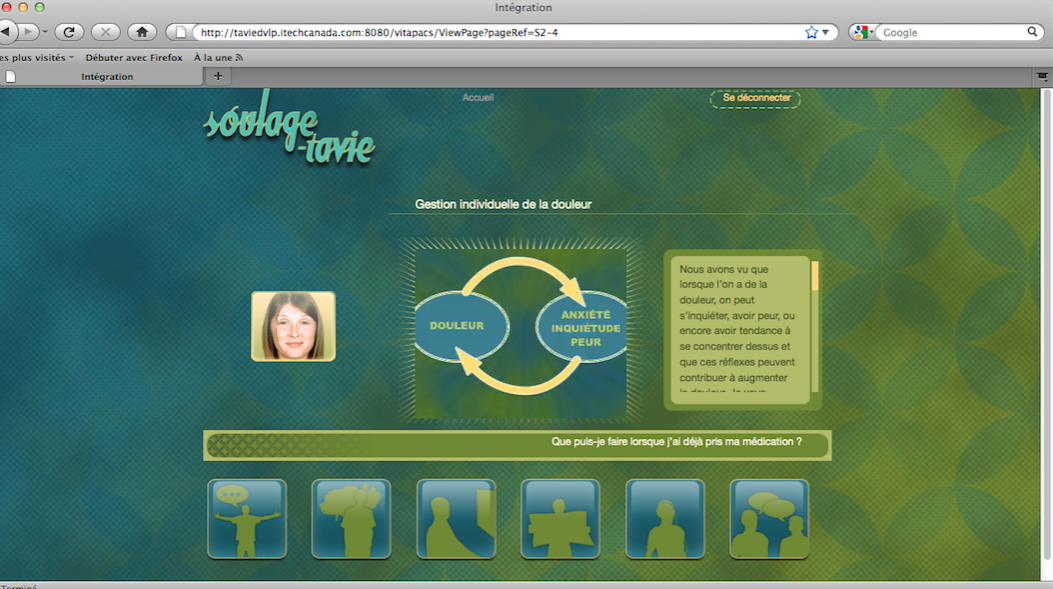
Animated integration page of the SOULAGE-TAVIE website displaying the nurse’s advice on pain and anxiety, and the patient’s navigation options for more information.

SOUtien à L’AutoGEstion-Traitement-Assistance Virtuelle Infirmière-Enseignement (SOULAGE-TAVIE), which translates into self-management, support treatment, virtual nursing assistance and education, is a computer-tailored and Web-based intervention developed for the self-management of pain after cardiac surgery [15]. Its home page is shown in Figure 1. This intervention begins with a brief, 5- to 10-minute screening of the patient’s pain-related beliefs and attitudes [16,17]. The second part consists of a 30-minute, tailored, preoperative session on a laptop computer facilitated by a virtual nurse that guides the participant through an animated, interactive learning process about the management of pain. The integration page for the website is shown in Figure 2. The information and strategies provided are specifically tailored to the participants’ profiles of pain beliefs and attitudes and are delivered according to a predetermined algorithm and to on-screen answers. Computer tailoring was offered as a complementary and personalized intervention to empower patients without adding a burden to patients and clinicians in the accelerated context of acute care.

A pilot randomized controlled trial (RCT) was conducted to examine the feasibility and preliminary effects of SOULAGE-TAVIE [18]. A total of 60 patients were randomly assigned to the experimental group (SOULAGE-TAVIE) and the control group (usual care including an educational pamphlet). Data were collected on admission and from day 1 through 7, postsurgery. Outcomes were pain intensity, pain interference with postoperative activities, individual pain barriers, pain catastrophizing, and analgesic consumption. Findings from this previous study provided initial evidence that the intervention was feasible and helpful in decreasing pain interference with a few postoperative activities, especially breathing and coughing. The intervention also influenced the way people coped with pain in modulating some individual barriers toward pain relief and opioid analgesic consumption. A brief questionnaire also revealed participants’ global satisfaction toward the intervention [15].

To our knowledge, SOULAGE-TAVIE is the first intervention of its kind in the acute care setting. Even though the feedback was positive, the results did not reveal the participants’ perceptions and preferences about the content and format of the intervention, and its acceptability remains unclear. Acceptability reflects the patients’ views of the intervention and can influence the implementation, patients’ adherence, and consequently, clinical outcomes [19,20]. Moreover, the experience of pain after cardiac surgery has been shown to be different between men and women [21-23]. The proportion of women is much lower in this population, therefore the experiences of male patients still influence our knowledge and interventions [23]. Research on Web-based and computer-tailored interventions is quite recent and because of its consistency with a patient-centered tailoring approach [11,14,24], a description of patients’ perceptions of SOULAGE-TAVIE’s acceptability would help illuminate its utility and value for the practice setting [19,25]. Therefore, the objectives of this study were (1) to describe SOULAGE-TAVIE’s acceptability from the perspective of adults experiencing pain after cardiac surgery and (2) to compare the perceptions of men and women.

## Methods

### Design

A mixed-method approach was used that captured both quantitative and qualitative data, but the latter were more heavily weighted (quan-QUAL). This approach captured the various attributes of patients’ perceptions of the intervention’s acceptability and compared the perceptions of men and women. Eligible patients were invited to complete a brief quantitative questionnaire to rate the acceptability of each intervention’s components before discussing each component in a semistructured interview with the interviewer.

### Sample

A quota sampling strategy was used to ensure adequate representation of men and women in the study sample. It is important to note that this study was undertaken in a different health setting and with a distinct sample than the previous pilot study. Because of feasibility issues, it was not possible to interview participants from the pilot RCT. Conducting an interview just before surgery or in the immediate postoperative phase would have been unrealistic considering the condition of the patients. The number of participants was guided by the principle of data saturation. However, 12 interviews are usually necessary to reach this point [26]. In total, 10 interviews with women and 10 interviews with men were conducted. The sample consisted of patients 21 years and older, who first had cardiac surgery involving a sternotomy—coronary artery bypass graft, valve replacement—within the past month. This time frame was selected since perceptions of the preoperative period and early postoperative pain experience would still be recent in mind, and would allow patients to participate in a 60-minute interview without causing undue fatigue. Moreover, the 6-week recovery period after cardiac surgery is recognized as challenging for patients, and pain is still very present [21,27].

### Procedure

A nurse asked eligible patients at the time of the follow-up—1 week after discharge—if they were interested in participating in the study. The nurse then communicated their contact information to the principal investigator (PI, GM), who called them to explain the study and arrange an interview within 1 month. Consent forms were signed at the time of the interviews. Semistructured interviews of approximately 60 minutes were conducted. Individual interviews were favored over focus groups to increase the feasibility of the study and to avoid group dynamics that could discourage the expression of divergent perceptions among participants [28]. Interviews took place in patients’ homes to avoid feasibility issues, such as coordinating the interview with the follow-up medical appointment or asking participants to travel for the interview during this recovery period. However, this choice was also methodological as location shapes interactions and relationships [29,30]. Choosing the participants’ homes could mitigate the traditional relationship between patients and health professionals and allow for opinions and preferences to be voiced.

The participants started by completing the sociodemographic and postoperative pain questionnaires, including presence of pain in the last month (yes/no), intensity pattern since discharge (decrease, increase, disappearance), and frequency in the last 7 days (continuous, occasional, absent). The interview began by using the tailored and Web-based intervention. After viewing the session, the participant was requested to rate each component in terms of its acceptability. Patients’ ratings of each component were used to solicit feedback on acceptability. The interviewer (GM) then invited each participant to comment on the acceptability of the intervention and on the need for, and nature of, modifying the components to fit their preferences.

### Instruments

The postoperative pain questionnaire is based on the Brief Pain Inventory (BPI) [31]. The BPI includes ten items: three items focus on pain intensity (0 for “no pain” to 10 for “worst possible pain”), and seven evaluate the impact of pain on general activity, mood, walking, work, relationships, sleep, and enjoyment of life. Participants were asked to base their ratings on their pain experience in the previous 7 days. Each item represents a subscale and can be scored and analyzed individually (0-10), with the anchors being “does not interfere” (0) and “completely interferes” (10). The internal consistency was supported (Cronbach alpha between .77 and .91) [31]. Some items were added in the context of the present study to measure the pain-related impact on appetite, concentration, and breathing/coughing. This version has been successfully validated with cardiac surgery patients [9,32] and was used by the investigator in a previous pilot study [18].

The intervention components were rated in terms of four attributes: (1) appropriateness in helping patients manage pain following cardiac surgery, (2) effectiveness in promoting pain management, (3) suitability, and (4) willingness to adhere, with the use of the treatment acceptability and preference (TAP) measure [33]. The ratings refer to a 5-point scale ranging from “not at all” (0) to “very much” (4). A total scale score between 0 and 4 was obtained as a mean of the scores from four items to reflect perceived intervention acceptability. The four items demonstrated internal consistency reliability (Cronbach alpha>.80) [33]. Three subitems were added to refine the rating, and consequently, the description of effectiveness, appropriateness, and suitability. The main author of the instrument validated the content of this adaptation. Table 1 presents the definitions of each acceptability attribute of the TAP measure according to Sidani et al [19].

An interview guide was developed and reviewed by two researchers (GM, MP) familiar with intervention and qualitative research. Table 2 shows questions from the semistructured interview guide.

**Table 1:** Definitions of acceptability attributes of the TAP measure [19].

Acceptability attribute^a^	Definition of attribute
Effectiveness	Perception of the extent to which the intervention is helpful in the short and long terms.
Appropriateness	Perception of the intervention’s overall reasonableness (ie, how logical).
Suitability	Judgment of the intervention’s intrusiveness and disruption in life (how easy, how long, etc).
Adherence	Extent to which patients are willing to follow or adhere to treatment.

^a^Acceptability is defined as the patients’ understanding of the treatment based on multiple elements.

**Table 2:** Semistructured interview guide adapted from the TAP measure [33].

Themes	Questions
Effectiveness	What do you find the most/least helpful about the computer-based program? In what way do you think the program would/would not have helped you manage your pain after surgery? In what way do you think the program would/would not have helped you decrease the impact of pain on your recovery?
Appropriateness	What do you find appropriate/not appropriate about the computer-based program? What strategies seem appropriate/inappropriate for managing postoperative pain? In what way are the strategies appropriate/not appropriate to pain management after surgery? What additional information (if any) would you like covered by the computer-based program?
Suitability	What pain management strategies in the computer-based program do you find suitable/not suitable? What do you think of the timing of the intervention? What do you think of the length of the intervention? What do you think of the virtual nurse?
Willingness to adhere	What is easy/not easy about using the computer-based program? What is easy/not easy about applying all the strategies? What (if anything) could be done to make the strategies easier to use? What (if anything) could be done to make the program more convenient to use?

### Data Analysis

Sociodemographic data and quantitative ratings from the BPI and the TAP questionnaire were analyzed descriptively. Frequency tables, medians, and ranges were used to summarize data for each item and to calculate the total score on the TAP measure. Data were not normally distributed (Shapiro-Wilk test<.05). Nonparametric Mann-Whitney U tests and chi-square tests were used to compare groups. Interviews were numerically recorded and transcribed by a qualified audio typist prior to content analysis following the approach by Miles and Huberman [34]. The QDA miner (Provalis Research) software was used to facilitate data management and organization of codes. The PI and a research assistant (RA) completed a 2-day training session on the use of this software. A preliminary generation of codes was based on the following attributes of acceptability highlighted by Sidani et al [19,33]: appropriateness, effectiveness, suitability, and willingness to adhere (see Table 1). Descriptive codes were created by attributing a code to each unit of analysis (words, phrases, or paragraphs). To ensure rigor and enhance credibility, separate coding (double coding) was conducted by the PI and the RA for the first 5 women and the first 5 men. Results were compared, and differences were discussed until a consensus was reached. However, no major differences were found. Additional codes (subcategories) were created when necessary. When a new code was generated, it was discussed as well. The PI kept a diary and noted questions or ideas and the discussions that occurred throughout the entire analysis process. Merging of similar descriptive codes created thematic categories representing a set of conceptual components. The content of each category and subcategory was compared between men and women. Frequency counts were also done to validate the emergence of a difference between the 2 subgroups.

## Results

### Sample Description

Eligible participants were recruited between November 2012 and May 2013. Characteristics of the sample are presented in Table 3. Participants ranged in age from 36 to 74 years (women, 36-74 years; men, 50-72 years). The majority of participants lived with a significant other except for 3 women and 2 men that were either divorced/separated or widowed but had a family member or a friend living close by. Of the 10 men, 7 had university degrees and 6 were still working. The chi-square test was significant for employment status (χ^2^_1_=4.9, *P*=.03). Most participants (16/20, 80%) had undergone a coronary artery bypass graft (CABG). It is noteworthy that almost 50% (9/20, 45%) of participants suffered from chronic pain—noncancer and noncardiac pain—with a median duration of 13 years for women (ranged from 72-360 months) and 3 years for men (ranged from 12-240 months). However, the Mann-Whitney U test was not significant for chronic pain duration (U=3.5, *P*=.11), meaning that there was no difference in chronic pain duration between women and men.

**Table 3:** Sociodemographic characteristics of participants.

Variables		Women (n=10)	Men (n=10)	Total (n=20)
Age in years, median (range)		67.0 (36-74)	65.5 (50-72)	66.5 (36-74)
**Marital status, n (%)**				
	Single	0 (0)	0 (0)	0 (0)
	Married or free union	7 (70)	8 (80)	15 (75)
	Separated/divorced/widowed	3 (30)	2 (20)	5 (25)
**Living arrangements, n (%)**				
	Lives with spouse (with or without children)	2 (20)	2 (20)	4 (20)
	Lives with family member or friend	5 (50)	6 (60)	11 (55)
	Lives alone	3 (30)	2 (20)	5 (25)
**Education level, n (%)**				
	Elementary school	1 (10)	0 (0)	1 (5)
	Middle school	5 (50)	2 (20)	7 (35)
	High school	2 (20)	1 (10)	3 (15)
	University	2 (20)	7 (70)	9 (45)
**Employment status, n (%)**				
	Full time/part time	2 (20)	6 (60)	8 (40)
	Retired	8 (80)	4 (40)	12 (60)
Presence of chronic pain, n (%)		4 (40)	5 (50)	9 (45)
Duration of chronic pain in months, median (range)		156.0 (72-360)	36.0 (12-240)	72.0 (12-360)
**Type of surgery, n (%)**				
	CABG	8 (80)	8 (80)	16 (80)
	Valve replacement (VR)	1 (10)	2 (20)	3 (15)
	CABG + VR	1 (10)	0 (0)	1 (5)

### Postoperative Pain Intensity and Interference

All patients had experienced postoperative pain following discharge in the previous month. Most participants (18/20, 90%) reported that the pain had decreased since discharge, except for 2 men who reported that it had resolved. In terms of pain frequency, 6 participants—3 men and 3 women—reported that pain was continuously present, 12 participants—6 men and 6 women—reported that it was occasionally present, and 2 men stated it was absent. Women reported a higher, but still mild, level of average pain (Table 4). Using the Mann-Whitney U test, a statistically significant difference was found solely for worst pain (U=19, *P*=.03). Women reported a higher intensity of worst pain, which was moderate compared to mild for men. However, when looking at pain interference with activities, women experienced less pain interference with breathing and coughing than men (U=20, *P*=.04).

**Table 4:** Median (minimum-maximum) of pain intensity and interference with activities in the last 7 days, according to the BPI.

Item	Women (n=10)	Men (n=10)	Total (n=20)	*P* value (Mann-Whitney U test)
Pain now	3.0 (0-5)	0.0 (0-6)	2.0 (0-6)	.31
Average pain	4.0 (2-5)	2.5 (0-4)	3.0 (0-5)	.08
Worst pain	6.0 (3-8)	3.5 (2-8)	5.0 (2-8)	.03
General activity	4.0 (0-9)	2.0 (0-8)	2.0 (0-9)	1.0
Mood	0.0 (0-5)	3.5 (0-7)	2.0 (0-7)	.40
Walking	0.0 (0-5)	0.0 (0-6)	0.0 (0-6)	.40
Work	2.0 (0-9)	0.0 (0-8)	0.0 (0-9)	.63
Relationships	0.0 (0-5)	0.0 (0-4)	0.0 (0-5)	.97
Sleep	4.0 (0-8)	1.0 (0-7)	1.0 (0-8)	.84
Enjoyment	0.0 (0-5)	0.0 (0-5)	0.0 (0-5)	.90
Appetite	0.0 (0-7)	0.0 (0-8)	0.0 (0-8)	.72
Concentration	0.0 (0-6)	0.0 (0-5)	0.0 (0-6)	1.0
Breathing/coughing	0.0 (0-6)	3.0 (0-8)	2.0 (0-8)	.04

### Acceptability of the SOULAGE-TAVIE Intervention

#### Overview

Participants’ total scores on the TAP measure indicated that SOULAGE-TAVIE is very acceptable. No difference was found in their overall appreciation. The results for each of the four attributes of the TAP measure is presented for men and women in Table 5, followed by a summary of the qualitative data that was generated from the interviews. The differences between men and women are described if necessary.

**Table 5:** Median (minimum-maximum) of ratings for intervention attributes according to the TAP measure.

Intervention attributes		Women (n=10)	Men (n=10)	Total (n=20)	*P* value (Mann-Whitney U test)
**Effectiveness**					
	How effective do you think the program would have been in helping you manage pain after cardiac surgery?	4.0 (3-4)	3.0 (2-4)	3.0 (2-4)	.06
	How effective do you think the program would have been in helping you decrease the impact of pain on your recovery?	3.0 (2-4)	3.0 (2-4)	3.0 (2-4)	.50
**Appropriateness**					
	How acceptable/logical does the program seem to you?	4.0 (3-4)	3.0 (2-4)	4.0 (2-4)	.008
	How appropriate does the program seem to be to help with pain management after surgery?	4.0 (3-4)	3.0 (2-4)	3.0 (2-4)	.28
**Suitability**					
	How easy does it seem to use the program?	4.0 (3-4)	3.5 (3-4)	4.0 (3-4)	.84
	How easy do you think it would have been for you to apply all strategies?	4.0 (3-4)	4.0 (3-4)	4.0 (3-4)	.90
**Willingness to adhere**					
	How willing would you have been to use the program?	4.0 (2-4)	4.0 (2-4)	4.0 (2-4)	.90
Total		3.6 (2.9-4.0)	3.3 (2.6-4.0)	3.4 (2.6-4.0)	.28

#### Experience of Raising Awareness

##### Overview

The first category that emerged after participants commented on their ratings of SOULAGE-TAVIE’s effectiveness and appropriateness was experience of awareness raising. Participants’ responses on the TAP measure in terms of effectiveness indicated that the intervention would have been very effective in helping them to manage their pain after surgery and in decreasing the impact of pain on their recovery. Women rated effectiveness slightly higher than men, although the difference was not statistically significant (U=22.5, *P*=.065). During the interviews, the benefits highlighted by participants were often related to satisfying their emotional needs, as reflected by the following interview excerpts: “What the nurse says is reassuring,” “You know what to expect,” and “It tones it down.” Informational benefits of SOULAGE-TAVIE were also highlighted by the following excerpts: “Before surgery you don’t necessarily ask the good questions,” “I would have known what to do,” and “You hear so many things from people before surgery, it gives you a clear answer.” Regarding appropriateness, participants rated the intervention as very logical and appropriate for pain management. A statistically significant difference was found for the question “How acceptable/logical does the program seem to you?” (U=13.5, *P*=.008), with women scoring higher than men. In the qualitative comments, appropriateness was related to the timing of the intervention and relevancy of the advice, as reflected by the comment “It would have been great to have this (intervention) before surgery.” Many participants stated, “All advice is important.”

Awareness raising emerged as both women and men outlined benefits that extended beyond information and reassurance. An awareness-raising experience was mentioned more often than emotional and informational benefits and was related to two subcategories: awareness raising toward pain beliefs and awareness raising toward pain management behaviors.

##### Experience of Awareness Raising Toward Pain Beliefs

When explaining how the intervention increased their awareness toward pain and its relief, women stated that they had been afraid to take pain medication, but now understood how pain medication could be helpful to their recovery, as reflected by the following excerpt: “I didn’t think I was so scared of pain medication.” However, men became aware that their beliefs about pain were dated and that it was important to relieve pain, as reflected by the following excerpts: “It’s normal to experience pain after surgery…you put your prejudices aside,” and “Often we have preconceptions…you end up punishing yourself.” Another man evoked his beliefs: “I wasn’t enough aware of the importance of relieving pain…I was educated the old way.”

##### Experience of Awareness Raising Toward Pain Management Behaviors

While referring to their experience of awareness toward pain beliefs, participants tended to reflect on their pain management behaviors. One man mentioned that the approach was innovative: “It makes you think…I didn’t have the right behavior toward pain—I would have acted differently.” Another man commented on his pain management behavior: “I tried to avoid moving too much.” A woman expressed her awareness-raising experience toward pain relief as follows: “Just to be aware that pain can be treated…it gives you the opportunity to be proactive. Sometimes I was waiting a long time before doing something.” Finally, all participants referred to the most important advice they had retained, which was almost always “to not put up with pain” or “to avoid peaks.” A good number of participants summarized the advice as “being preventive toward pain.”

#### Experience of Convenient Support

##### Overview

The second category that emerged after having participants comment on their rating of SOULAGE-TAVIE’s suitability was experience of convenient support. Participants reported that the intervention was very suitable. The program was rated as very easy to use, and the strategies were rated as very easy to apply. Ratings on the TAP measure were almost identical for women and men. When commenting, more men than women reported that they were comfortable with the Web-based delivery mode. Some women were a bit reticent toward the use of the computer at the beginning of the interview but evolved during the demonstration and discussion, as reflected by the following interview excerpts: “It would have been a bit difficult at the beginning but I would have managed…I could have asked my grandson,” “I don’t use computers but I would have tried,” and “Now that you showed it to me it’s not complicated.” The majority of participants commented on suitability in terms of adequate length, timing, and, especially, delivery mode. The use of a Web-based application as a delivery mode was perceived as a logical fit with today’s living, as reflected by the following excerpts: “Everyone goes on the Web to look for information,” “It’s a new era,” and “Nowadays, computers are essential.” Two subcategories emerged while describing their experience of support: flexibility and interaction.

##### Experience of Flexible Support

Flexibility in terms of personal readiness was the most reported advantage of using a Web application, as reflected by the following excerpt: “You can use it at your convenience…with a clear head.” Flexibility was also illustrated in terms of improved access to information in the context of acute care by the following excerpts: “You can go back anytime,” and “If the nurse could not take the time to explain, I’d still have this information.”

##### Experience of Interactive Support

The virtual nurse was another aspect raised with regard to the delivery mode. A new category was then added as participants identified the benefits of interacting with the virtual nurse. Participants described two aspects related to the interaction. First, they perceived they were more attentive and they had a better retention of the information as reflected by these excerpts: “It’s easier to understand when you listen to the nurse,” “You are more focused…sometimes, when you read you don’t do it right, you want to finish quickly,” and “She catches your attention.” The other aspect is more related to the relationship with the nurse: “It’s more friendly,” “I find it more personalized…you feel like you’re talking to someone,” “It’s not like being in front of a real nurse but it’s more human…it breaks the ice,” “I trust her,” and “It adds some authenticity to the advice.”

#### Experience of Guidance

The third category that emerged after having participants comment on their ratings of their willingness to adhere to SOULAGE-TAVIE was experience of guidance. Participants’ scores on the TAP measure for men and women indicated that they were very willing to use the Web application. Similarly, during the interviews they insisted that they would have liked to use the Web application before their surgery: “It would have been useful,” “It would have been good for me,” and “It’s a good program.” Men and women were very satisfied with their experience with the intervention. It is noteworthy that participants tended to refer to the program as a “good guide” allowing for self-determination and control: “Advice remains advice, it is not an obligation…you cannot take people by the hand.” Men expressed their willingness to adhere to the intervention by pointing out the suitability of the intervention: “It’s a useful tool…very practical.” Women tended to focus on the importance and relevance (appropriateness) of the advice for other patients facing pain after cardiac surgery: “Necessary advice that everyone should get,” and “It’s important that everyone knows.” Table 6 summarizes the content analysis for both women and men.

**Table 6:** Content analysis summary for women and men.

Attributes of the TAP measure	Category	Subcategory	Representative quotes from groups, verbatim
Effectiveness: In helping manage pain In helping decrease pain impact	Awareness raising	Toward pain beliefs	Women, on medication reluctance: “I didn’t think I was so scared of pain medication.” Men, on pain normalization: “Often we have preconceptions…you end up punishing yourself.”
Appropriateness: Logical/acceptable Appropriate to help with pain management	Awareness raising	Toward pain management behavior	Both: “It makes you think…I didn’t have the right behavior toward pain, I would have acted differently.” Both: “Just to be aware that pain can be treated…it gives you the opportunity to be proactive—sometimes I was waiting a long time before doing something.”
Suitability: Easy to use Easy to apply	Convenient support	Flexible	Both, on readiness: “You can use it at your convenience…with a clear head.” Both, on access: “You can go back anytime.”
		Interactive	Both, on attention: “You are more focused.” Both, on interaction: “It’s more friendly.”
Willingness to adhere	Guidance	Self-determination	Both: “It’s a good guide.” Both: “You can’t take people by the hand." Women, on awareness raising: “It’s important that everyone knows.” Men, on convenient support: “It’s a useful tool…very practical.”

## Discussion

### Principal Findings

The aim of this study was to provide a thorough description of the acceptability of a Web-based intervention for the self-management of pain after cardiac surgery and to delineate potential differences between the perceptions of women and men about the acceptability of the intervention. Based on the TAP scores, the intervention was perceived as very acceptable by both groups. Although the sample was quite small, it was observed that women rated the intervention higher in terms of appropriateness than men. Women’s higher postoperative pain intensity scores and longer duration of chronic pain may have resulted in greater support needs and, consequently, they experienced more satisfaction with the intervention. This difference might also be related to psychosocial characteristics. It was previously observed that women experienced emotional distress while waiting for surgery and after discharge and that they sought to “preserve the self” and accept their modified health and functional status [35-37]. Moreover, in the current study more women were retired than men. Hence, the mixed-method approach allowed for a better understanding of the TAP measure scores—total and specific attributes—since they were high and quite homogenous across men and women.

Overall, the participants indicated that in addition to the emotional and informational benefits, the SOULAGE-TAVIE intervention would have generated an experience of increased awareness of their own pain beliefs and pain management behaviors as well. Furthermore, men and women underlined that they would have acted differently in the face of pain. This finding is very interesting because it validates the appropriateness of the underlying therapeutic strategy and the content of messages, and demonstrates the acceptability of the method for patients experiencing postoperative pain after cardiac surgery. Indeed, the intervention development was based on the Elaboration Likelihood Model (ELM), which focuses on imparting information that stimulates reflection and a change in attitude [38,39]. If motivated, individuals are active information processors as they can carefully consider messages, and relate them to other information and to their own experiences [39-41]. The success of this strategy relies on the selection of appropriate tailoring variables that will enhance message relevance (ie, pain belief and attitudes) but also influence the targeted behavior (eg, pain management). Hence, the use of theory and the combination of behavioral change techniques were associated with an increase in impact by Web-based interventions [12]. The only difference between women and men was the nature of this awareness raising. Women reported awareness about their reluctance to use medication and tendency to wait too long to use pain medication. This is consistent with previous work that found women wanted to take as little medication as possible and did not follow the recommendations despite reporting high levels of pain [22]. Men reported awareness about their preconceptions and normalization of pain, which led to the same behavior of procrastinating before relieving their pain. In fact, the computer tailoring method, which screens for pain beliefs and attitudes before message delivery, addresses this gender difference regarding awareness.

The second main result is that participants would have experienced a convenient support. Men and women judged the Web-based intervention as suitable because of its flexibility. Indeed, Web-based, tailored interventions seem to offer both more control to patients in terms of content and timing [14,24], and more outreach when access to care is limited [42,43]. According to the participants, the Web-based delivery seemed to include another advantage over a more traditional printed format: increased attention and retention. They found it was easier to integrate the information and stay focused. Indeed, Web-based tailored interventions provide greater interactivity and may result in more engagement [14]. In fact, the virtual nurse personified interactivity in SOULAGE-TAVIE and personal contact tends to support behavior change in Web-based interventions [12]. Not only does the use of information technologies allow messages to be more attractive [24], but it also seems to enhance cognitive processing through customization [44,45]. An experience of guidance accompanied by self-determination rounded out their overall appreciation of the intervention. This result underlines the acceptability of the general approach of SOULAGE-TAVIE in terms of user control—self-guided/automated as opposed to expert led/directed—for this population [24]. This type of approach is usually privileged with brief interventions [24]. Finally, it is noteworthy that men expressed their willingness to use or adhere to the intervention due to format suitability and practicality, whereas women cited the appropriateness of advice. Until now, gender has not been shown to make a difference in terms of interest in Web-based, delivered interventions [43] or effect size [11,14]. Nevertheless, education might have played a role in this difference of perception considering that fewer women had a university level of education. Indeed, it was previously demonstrated that lower-educated individuals showed higher attention and processing of information which led to greater intention to use the website [46].

### Limitations and Future Directions

As with other studies, this study had some limitations. The participants were recruited approximately one month after their cardiac surgery. They were not in a preoperative frame of mind, which may have influenced their perceptions with regard to effectiveness and appropriateness. However, the recent experience of surgery and postoperative pain may have contributed to the perceived relevance of the intervention. Also, more women were retired and could have suffered from social isolation, which could contribute to the appreciation of the intervention and the study participation.

However, the participants’ experience with the intervention during the recovery phase revealed the relevance of the intervention before surgery and after discharge as well. Previous studies on pain after cardiac surgery following discharge highlighted high pain levels and the contribution of pain beliefs and attitudes to pain levels, especially in women [21,22]. Authors highlighted the relevance of an intervention initiated before surgery and extended after discharge [47]. Moreover, the delivery of information at discharge seems to face the same issues as the delivery of information before surgery [47]: patients have little time to integrate content. Thus a Web-based intervention could be a good option to face the challenges experienced both preoperatively and during the recovery period. Considering that SOULAGE-TAVIE has demonstrated potential effects on the pain experience in the first week after surgery, it would be interesting to assess its impact after discharge and explore the possibility of preventing chronic pain after cardiac surgery or at least disability. Moreover, SOULAGE-TAVIE targets psychosocial characteristics that are included in models looking at the transition from acute to chronic postoperative pain [5,48].

### Conclusions

When designing a new intervention, the mixed-method approach and data triangulation are very useful as they capture patients’ perceptions and the mechanisms underlying the effectiveness of intervention. This study described the acceptability of computer tailoring and persuasive communication to modulate pain beliefs and attitudes in an acute care context. Participants perceived the intervention to be very acceptable in terms of content and format, although women and men differed in their reasons for its acceptability. Women were willing to adhere to the intervention based on the importance and relevance of the advice provided whereas men were more focused on the delivery mode and their appreciation of its flexibility. The participants’ experience of SOULAGE-TAVIE after discharge revealed its relevance across the continuum of care. This approach seems to be an interesting avenue to influence the transition from acute to chronic postoperative pain.

## Abbreviations

BPI: Brief Pain Inventory
CABG: coronary artery bypass graft
CPOP: chronic postoperative pain
ELM: Elaboration Likelihood Model
PI: principal investigator
RA: research assistant
RCT: randomized controlled trial
SOULAGE-TAVIE: SOUtien à L’AutoGEstion-Traitement-Assistance Virtuelle Infirmière-Enseignement
TAP: treatment acceptability and preference
VR: valve replacement
